# Risk prediction of patients with perioperative pneumonia exacerbation being transferred to the ICU based on different machine learning methods

**DOI:** 10.3389/fmed.2026.1910537

**Published:** 2026-08-14

**Authors:** Xiao Chen, Boyi Zhang, Dan Wang, Jiale Li, Binbin Chen, Yifei Liu, Xiaojing Zhou, Jing Du, Na Li

**Affiliations:** 1 Department of Anesthesiology, Hainan Affiliated Hospital of Hainan Medical University (Hainan General Hospital), Haikou, Hainan, China; 2 Department of Anesthesiology, Hainan General Hospital (Hainan Affiliated Hospital of Hainan Medical University), Haikou, Hainan, China; 3 Department of Anesthesiology, Hainan Women and Children's Medical Center (School of Pediatrics, Hainan Medical University), Haikou, Hainan, China

**Keywords:** intensive care unit, machine learning, perioperative period, pneumonia, risk prediction

## Abstract

**Objective:**

To develop a prediction model for the risk of patients with perioperative pneumonia exacerbation being transferred to the intensive care unit (ICU) based on different machine learning algorithms and to evaluate its predictive efficacy.

**Methods:**

A total of 309 surgical inpatients (2022–2025) were included; 80 (25.89%) were transferred to the ICU due to exacerbation of pneumonia. Patients were randomly divided in a 7:3 into training (217) and validation (92) sets. The least absolute shrinkage and selection operator (LASSO) regression was applied to identify predictors of ICU transfer following pneumonia exacerbation. Four models (RF, SVM, LR, and GBM) were constructed to predict ICU transfer in patients with perioperative pneumonia exacerbation. Performance was assessed using receiver operating characteristic (ROC) curves, area under the curve (AUC), calibration curves, and decision curve analysis (DCA). The Shapley additive explanation (SHAP) method was used to explain the contribution of the variables to the optimal model's predictions.

**Results:**

The LASSO selected surgery type, surgery duration, ASA class, and WBC count. Among the four models, the SVM model achieved the highest AUC in the validation set (0.817), with a training set AUC of 0.862. The calibration was good, and the DCA showed a net clinical benefit. SHAP confirmed that all four were key predictors. Two cases illustrated model use: an ICU-transferred patient had a SHAP score of 0.897, while a non-transferred patient scored 0.073.

**Conclusion:**

In this study, four machine learning models were developed and validated to predict ICU transfer in patients with perioperative pneumonia exacerbation. The SVM model showed the highest validation AUC among the four models. WBC count, duration of surgery, ASA grade, and type of surgery were identified as key risk factors for progression from pneumonia exacerbation to ICU admission. However, as a single-center study with internal validation only, external validation is required before clinical application.

## Introduction

Postoperative pneumonia (POP) is a common serious complication of clinical anesthesia and surgical practice. In patients with community-acquired pneumonia (CAP), the original pneumonia is prone to worsen under the combined stress factors of surgical trauma, anesthetic drugs, tracheal intubation, and postoperative pain, which can subsequently lead to respiratory failure, septic shock, and multiple organ dysfunction (1). Studies have shown that POP accounts for approximately 50% of all hospital-acquired pneumonia cases, with an incidence rate of 1.5% to 15.8%, and is closely related to prolonged hospital stay, increased medical costs, and significantly increased mortality (2, 3). For patients with perioperative pneumonia exacerbation, timely identification of the risk of transfer to the intensive care unit (ICU) is crucial for optimizing medical resource allocation and implementing early preventive interventions.

Traditional perioperative risk assessment mainly relies on clinical experience and classic scoring systems, such as the American Society of Anesthesiologists Physical Status Classification (ASA-PS) and EuroSCORE for cardiac surgery (4). However, these traditional scoring tools have significant limitations. For instance, they are mostly based on limited variables and are difficult to comprehensively capture the complex multi-factor interactions during the perioperative period. Simultaneously, traditional scoring systems are mostly linear models and cannot effectively handle the generally non-linear relationships between clinical variables. Moreover, these tools provide generalized risk stratification and lack precise predictive ability for individuals (5). A recent systematic review and meta-analysis included 123 studies covering 116 POP complication prediction models. The results showed that although 50% of the models reported good discrimination (c-statistic > 0.8), up to 90.2% of the models had high bias risk, and most of the models lacked external validation, and their clinical practicality remains unclear (6).

In recent years, with the improvement of medical informatization levels and the widespread adoption of electronic medical record systems, the accumulation of massive clinical data has provided a foundation for the innovation of risk prediction models (7). Machine learning (ML) technology, due to its powerful self-learning ability and pattern recognition capability, demonstrates unique advantages in the field of medical risk prediction (8). Compared with traditional statistical methods, machine learning algorithms can automatically identify non-linear relationships and feature interactions in high-dimensional data, and achieve dynamic optimization of the model through continuous learning of new data (9). Bellini et al. (4) conducted a systematic review that included 36 perioperative machine learning prediction model studies from 2015 to 2021, and the results showed that machine learning algorithms demonstrated good efficacy in predicting postoperative mortality, ICU admission, respiratory system complications and acute kidney injury. The area under the receiver operating characteristic curve (AUC) of gradient boosting and random forest exceeded 0.90, significantly outperforming traditional scoring tools.

Regarding the risk of patients with perioperative pneumonia exacerbation being transferred to the ICU, there is currently a lack of systematic comparative studies on the predictive efficacy of different machine learning algorithms. In addition, most existing prediction models focus on the risk of POP (2), few studies on the prediction of the progression of patients with exacerbated pneumonia to the point where ICU support is required. This study aimed to use clinical real-world data to compare the performance of four algorithms, namely Logistic Regression (LR), Random Forest (RF), Support Vector Machine (SVM), and Gradient Boosting Machine (GBM), in predicting the risk of patients with perioperative pneumonia exacerbation being transferred to the ICU. It aims to select the optimal model and identify key predictive factors to provide quantitative evidence for the early identification of high-risk patients and timely adjustment of perioperative management strategies in clinical practice.

## Materials and methods

### Materials

A total of 309 inpatients who underwent surgical procedures in our hospital from January 2022 to December 2025 were selected. The inclusion criteria were as follows: (1) A clear diagnosis of CAP was made before surgery based on the “Guidelines for the diagnosis and treatment of adult community acquired pneumonia in China (2016 Edition)” (10); (2) Existing CAP worsened within 24 h after surgery; and (3) age ≥ 18 years. Exclusion criteria were as follows: (1) already admitted to the ICU before surgery; (2) Complicated with severe coagulation dysfunction; (3) Died during operation; (4) Clinical data missing by more than 20%. This retrospective study was approved by the Medical Ethics Committee of Hainan General Hospital (Approval Number: Medical Research [2025]128). The requirement for written informed consent was waived due to the retrospective nature of the study.

### Clinical data

The following variables were collected through the hospital electronic medical record system (EMR) and anesthesia information system: demographic characteristics including age, gender, marital status (divorced, widowed, unmarried, and married); preoperative factors including ASA classification (I, II, III, IV), smoking history, underlying diseases (hypertension, diabetes, respiratory disease history), white blood cell count (WBC), direct bilirubin, fibrinogen, CRP, blood glucose, neutrophils, lymphocytes, globulin, absolute value of monocytes, and hemoglobin. Intraoperative factors included cardiothoracic surgery, surgical type (emergency/elective), surgical grade (grade 1, 2, 3, 4), surgical duration ( ≤ 120 min, 120–180 min, 180–240 min, > 240 min), general anesthesia, classification of anesthesia time ( ≤ 120 min, 120–180 min), (180 to 240 min, > 240 min), and intubation. Postoperative factors included death (yes/no).

### Grouping situation

The primary outcome of this study was postoperative ICU admission due to exacerbation of pneumonia. Accordingly, patients were classified into the ICU admission group and the non-ICU admission group. ICU admission decisions were based on standardized intensive care admission criteria (11–15). Following consultation with the intensive care team, patients were admitted to the ICU if they met any of the following criteria: (1) Respiratory failure: requiring non-invasive positive pressure ventilation (NPPV) or endotracheal intubation with mechanical ventilation, or a progressive decline in the oxygenation index (PaO_2_/FiO_2_) to < 300 mmHg; (2) Hemodynamic instability: requiring continuous infusion of vasoactive agents (e.g., norepinephrine ≥0.1 μg/kg/min) to maintain a mean arterial pressure (MAP) ≥65 mmHg; (3) Sepsis or septic shock: an increase in the Sequential Organ Failure Assessment (SOFA) score of ≥2 points from baseline; (4) Impaired consciousness: a Glasgow Coma Scale (GCS) score ≤ 12; or (5) Other conditions requiring intensive care monitoring and treatment: including persistent severe acidosis (pH < 7.2) or coagulopathy requiring continuous blood product transfusion, with ICU admission determined jointly by the attending physician and the ICU physician. All ICU admission decisions followed these standardized criteria to minimize selection bias arising from variations in physician judgment or ICU bed availability.

### Data preprocessing

To ensure the reliability of the model evaluation, all data preprocessing was performed after dataset partitioning. First, the dataset was randomly divided into a training set (217 cases) and a validation set (92 cases) in a 7:3 ratio. Among the 309 included patients, the proportion of missing values for all 26 candidate variables was < 5%, with an overall missing rate of < 5%. Missingness was predominantly observed in the white blood cell count (1.6%), duration of surgery (1.0%), and BMI (0.6%), whereas all other variables had missing rates below 1%. Missingness was assumed to be missing at random (MAR). Multiple imputations were performed using the fully conditional specification method with 10 imputations. Predictive mean matching was used for continuous variables and logistic regression for binary variables. Multiple imputation was performed exclusively on the training set, with imputation parameters estimated from the training data and applied to the validation set to prevent information leakage. Continuous variables were standardized using Z-score normalization, and categorical variables were one-hot encoded, with all parameters estimated exclusively from the training set and applied to the validation set. All feature selections were performed on the training set, and the validation set was used solely for the final model evaluation.

### Model construction and validation

To address the issue of class imbalance, the SMOTE algorithm was employed to oversample minority class samples during the model training process. The specific procedure was as follows: (1) hyperparameter tuning of the model was performed using five-fold cross-validation on the training set. SMOTE was only applied within the training subset of each fold, and no oversampling was performed on the validation subset to prevent the synthesized samples from leaking into the validation fold; (2) the optimal hyperparameters were used to train the final model on the training set that had been balanced by SMOTE; and (3) the model performance was evaluated on an independent validation set, which did not undergo any oversampling. This process ensured that all performance evaluations were based on samples independent of the model training process, avoiding optimistic estimates due to data leakage.

Logistic Regression (LR) used stepwise regression and selected variables based on AIC. RF was set with n_estimators = 500, max_depth = 10, and min_samples_leaf = 2. It was tuned using a grid search and five-fold cross-validation. GBM was set with learning_rate = 0.1, max_depth = 6, subsample = 0.8, and colsample_bytree = 0.8, and scale_pos_weight was set as the reciprocal of the positive and negative sample ratio. It was tuned using five-fold cross-validation. SVM used the RBF kernel and optimized the penalty parameter C (0.1–100) and the kernel parameter γ (0.001–1) through five-fold cross-validation. All model parameter tuning and training were strictly limited to the training set, and the validation set was only used for the independent evaluation of the final model.

### Statistical analysis process

Data analysis was performed using SPSS version 27.0 and R version 4.1.3. Continuous variables with a normal distribution were expressed as mean ± standard deviation (SD), and between-group comparisons were performed using the independent-samples *t* test. Continuous variables with a non-normal distribution are expressed as median (interquartile range) [M (Q1, Q3)] and compared using the Mann–Whitney U test. Categorical variables are expressed as number (percentage) [*n* (%)] and compared using the χ^2^ test or Fisher's exact test, as appropriate. A two-sided *P* value < 0.05 was considered statistically significant.

The Least Absolute Shrinkage and Selection Operator (LASSO) regression was used for feature selection in the training set, and the optimal penalty parameter (λ) was determined using ten-fold cross-validation. Variables with non-zero coefficients were retained as predictive features. Based on these selected features, four machine learning models were developed: LR, RF, GBM, and SVM. Before model training, the training set was balanced using SMOTE. Hyperparameter tuning was performed using five-fold cross-validation. To assess model stability, bootstrap optimism correction was performed with 200 resamples, where each bootstrap sample was drawn with replacement from the training set, and the optimism-corrected AUC was calculated based on the difference between the AUC in the bootstrap sample and the out-of-bag sample. Model performance was evaluated using the area under the receiver operating characteristic curve (AUC), accuracy, sensitivity, specificity, precision, F1 score, calibration slope, and Brier score. Calibration curves were used to assess model calibration, whereas Decision Curve Analysis (DCA) was performed to evaluate the clinical net benefit of each model. Finally, SHapley Additive exPlanations (SHAP) were used to interpret the best-performing model and quantify the contribution of each feature to the prediction results. The final hyperparameters for each machine learning model were as follows: RF was configured with ntree = 500 and mtry = 2 (the square root of the number of features); SVM employed the RBF kernel, with the optimal penalty parameter C = 1 and kernel parameter γ = 0.1 determined by five-fold cross-validation; GBM was configured with learning_rate = 0.05 and interaction.depth = 3, with the optimal number of iterations determined by five-fold cross-validation; LR used the penalty parameter λ determined by ten-fold cross-validation. This study was conducted in accordance with the TRIPOD+AI (Transparent Reporting of a multivariable prediction model for Individual Prognosis Or Diagnosis–Artificial Intelligence) reporting guidelines.

## Result

### Comparison of clinical data between the training set and the validation set

In this study, 309 subjects were randomly divided into the training set (*n* = 217) and the validation set (*n* = 92) in a 7:3 ratio. Among the 217 patients in the training set, 54 cases (24.88%) were transferred to the ICU. Among the 92 cases in the validation group, 26 cases (28.26%) were transferred to the ICU. There were no statistically significant differences in the clinical data between the training and validation groups (*P* > 0.05, Table 1).

**Table 1:** Comparison of clinical data between the training set and the validation set.

Parameters	Training set (*n* = 217)	Validation set (*n* = 92)	t/χ^2^	*P*
Sex [*n* (%)]			0.161	0.688
Male	128 (58.98)	52 (56.52)		
Female	89 (41.01)	40 (43.48)		
Age (years)	61.39 ± 14.62	61.53 ± 13.12	0.336	0.737
BMI (kg/m^2^)	22.51 ± 3.44	22.66 ± 3.38		
Marriage [*n* (%)]			5.221	0.156
Divorce	4 (1.84)	1 (1.09)		
Widowed	9 (4.15)	0 (0.00)		
Unmarried	8 (3.69)	6 (6.52)		
Married	196 (90.32)	85 (92.39)		
Cardiac surgery [*n* (%)]			1.449	0.229
Yes	54 (24.88)	29 (31.52)		
No	163 (75.12)	63 (68.48)		
Type of surgery [*n* (%)]			0.336	0.562
Emergency surgery	21 (9.68)	7 (7.61)		
Elective surgery	196 (90.32)	85 (92.39)		
Surgical grading [*n* (%)]			1.659	0.646
1	1 (0.46)	1 (1.09)		
2	6 (2.76)	2 (2.17)		
3	75 (34.56)	26 (28.26)		
4	135 (62.21)	63 (68.48)		
Duration of surgery [*n* (%)]			3.275	0.351
≤ 120 min	57 (26.27)	18 (19.57)		
120 min-180 min	74 (34.10)	28 (30.43)		
180 min-240 min	36 (16.59)	21 (22.83)		
>240 min	50 (23.04)	25 (27.17)		
Endotracheal intubation [*n* (%)]			0.074	0.786
Yes	174 (80.18)	75 (81.52)		
No	43 (19.82)	17 (18.48)		
General anesthesia [*n* (%)]			0.012	0.913
Yes	192 (88.48)	81 (88.04)		
No	25 (11.52)	11 (11.96)		
ASA grade [*n* (%)]			0.780	0.854
I	3 (1.38)	1 (1.09)		
II	162 (74.65)	64 (69.57)		
III	41 (18.89)	20 (21.74)		
IV	11 (5.07)	3 (3.26)		
Anesthesia duration [*n* (%)]			3.484	0.323
≤ 120 min	63 (29.03)	23 (25.00)		
120 min−180 min	57 (26.27)	26 (28.26)		
180 min−240 min	35 (16.13)	22 (23.91)		
>240 min	44 (20.28)	21 (22.83)		
Hypertension [*n* (%)]			0.062	0.803
Yes	62 (28.57)	25 (27.17)		
No	155 (71.43)	67 (72.83)		
Diabetes mellitus			0.032	0.857
Yes	29 (13.36)	13 (14.43)		
No	188 (86.64)	79 (85.87)		
History of respiratory disease [*n* (%)]			1.576	0.209
Yes	9 (4.15)	7 (7.61)		
No	208 (95.85)	85 (92.39)		
Postoperative death			0.018	0.892
Yes	2 (0.92)	1 (1.09)		
No	215 (99.08)	91 (98.91)		
WBC ( × 10^9^/L)	7.82 (6.11, 10.06)	7.57 (6.03, 10.65)	0.045	0.964
Direct bilirubin (μmol/L)	3.10 (2.33, 4.35)	3.39 (2.60, 4.94)	1.921	0.055
Fibrinogen (g/L)	3.42 (2.85, 4.25)	3.36 (2.71, 4.27)	0.536	0.592
CRP (mg/L)	8.36 (2.65, 25.32)	5.56 (2.14, 23.97)	1.333	0.182
FBG (mmol/L)	5.22 (4.81, 6.09)	5.23 (4.88, 6.39)	0.219	0.827
Neutrophil percentage (%)	4.84 (3.39, 7.40)	4.88 (3.38, 8.22)	0.322	0.747
Lymphocyte percentage (%)	1.68 (1.27, 2.05)	1.62 (1.18, 2.20)	0.201	0.841
Globulin (g/L)	30.50 ± 4.32	30.05 ± 4.54	0.826	0.409
Absolute monocyte count ( × 10^9^/L)	0.52 (0.39, 0.71)	0.50 (0.37, 0.67)	1.267	0.205
Hemoglobin (g/L)	115.65 ± 22.81	116.44 ± 27.49	0.206	0.737

ASA, American Society of Anesthesiologists Physical Status Classification System; WBC, white blood cell; CRP, C-reactive protein; FBG, fasting blood glucose.

### Feature variable selection

**Figure 1:**
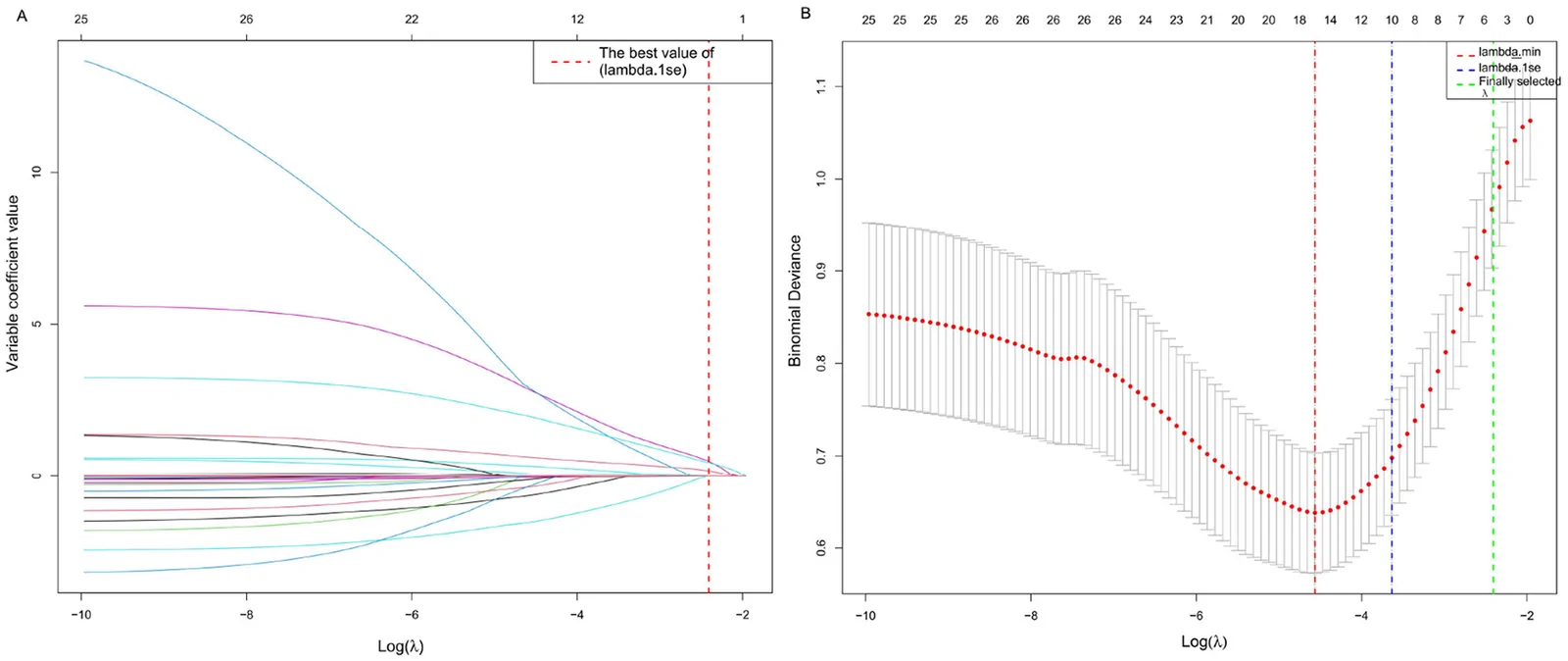
Variable screening using LASSO regression analysis. **(A)** Dynamic plot of variable selection in LASSO regression analysis; **(B)** 10-fold cross-validation plot for LASSO regression analysis; LASSO, least absolute shrinkage and selection operator.

Based on the training set data, with ICU admission as the dependent variable and each indicator in Table 1 as independent variables, the optimal penalty coefficient λ was determined using 10-fold cross-validation in the LASSO regression model. Vertical lines were drawn at λ and λ+SE, respectively. At λ+SE, four indicators with non-zero LASSO regression coefficients were ultimately selected: surgery type, surgery duration, ASA grade, and white blood cell count (Figure 1, Table 2).

**Table 2:** Variable screening in LASSO regression analysis.

Factors	Variable coding	λ+1se
Surgery type (Emergency)	Emergency = 1, Scheduled = 0	0.494
Surgery duration	≤ 120 min = 1, 120~180 min = 2, 180~240 min = 3, >240 min = 4	0.177
ASA grade	I = 1, II = 2, III = 3, IV = 4	0.467
WBC	Entry of original value	0.004

ASA, American Society of Anesthesiologists Physical Status Classification System; WBC, white blood cell; LASSO, least absolute shrinkage and selection operator.

### Comparison of predictive performance of machine learning models

Based on the four feature variables selected by LASSO regression in the training set, four machine learning models, namely RF, SVM, LR, and GBM, were constructed. The results showed that all four models exhibited good predictive performance in the training set. Among them, the RF model achieved the highest AUC of 0.981 (95% CI: 0.968 ~ 0.994), followed by the GBM model with an AUC of 0.918 (95% CI: 0.883 ~o 0.954). In contrast, both the SVM and LR models had an AUC of 0.862 (95% CI: 0.809–0.915 and 0.808–0.916, respectively).

In the validation set, the SVM model showed the highest AUC among the four models, with an AUC of 0.817 (95% CI: 0.727 ~ 0.907), a sensitivity of 0.308, a specificity of 0.955, an accuracy of 0.772, and an F1 score of 0.432. DeLong test showed that the AUC difference between SVM and LR (0.817 vs. 0.794) was not statistically significant (*P* = 0.110). Although the RF model achieved the highest AUC (0.981) in the training set, its AUC decreased to 0.745 in the validation set, suggesting potential overfitting. The LR and GBM models achieved validation set AUCs of 0.794 (95% CI: 0.702 ~ 0.887) and 0.766 (95% CI: 0.667 ~ 0.865), respectively.

**Figure 2:**
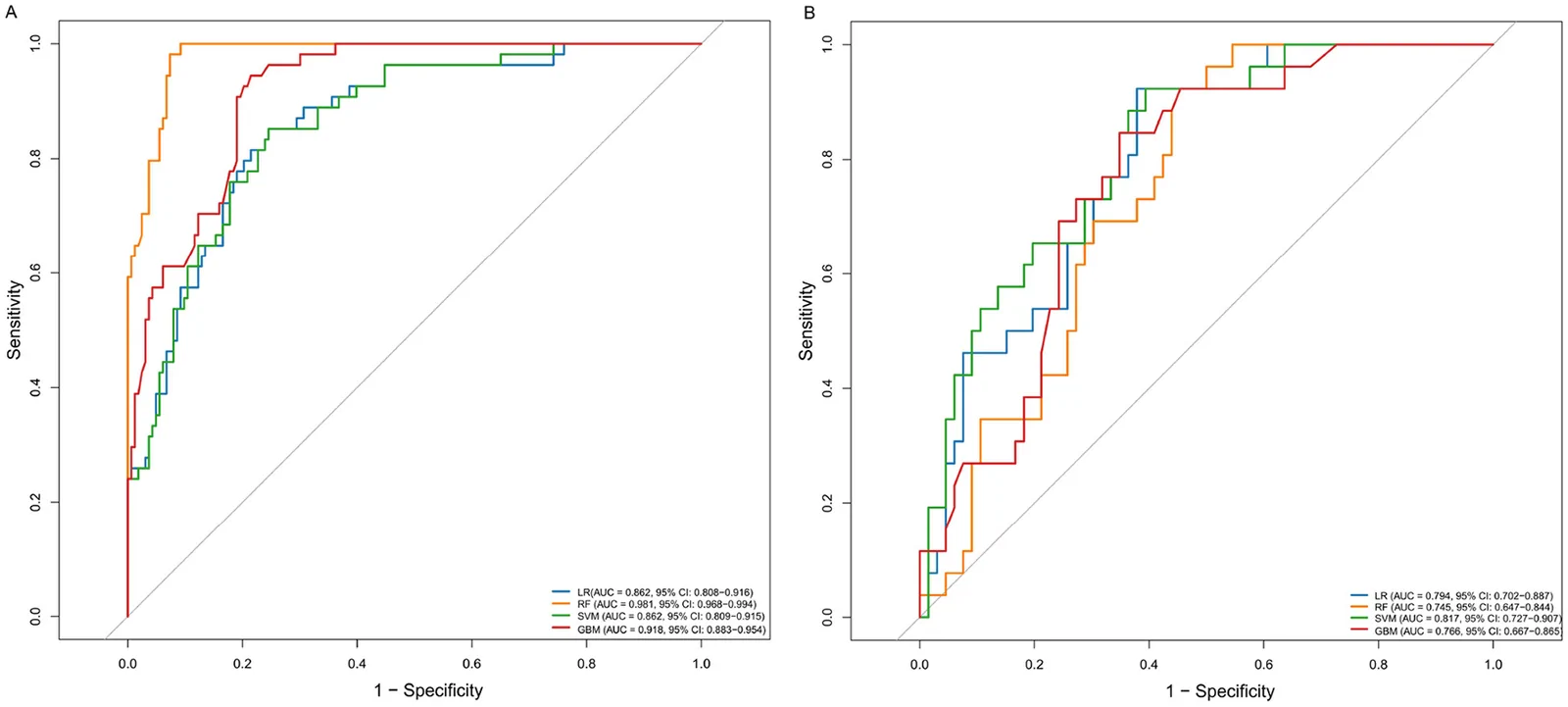
ROC curves of different machine learning models for predicting ICU admission in patients with perioperative pneumonia exacerbation; **(A)** AUC of the four models in the training set; **(B)** AUC of the four models in the validation set; RF, random forest; SVM, support vector machine; LR, logistic regression; GBM, gradient boosting machine; LASSO, least absolute shrinkage and selection operator.

In summary, the SVM model showed the numerically highest AUC in the validation set among the four models. However, the improvement over LR was modest (Δ = 0.023) and not statistically significant (*P* = 0.110). The model may serve as a supportive reference for clinical decision-making, but further external validation is needed to confirm its generalizability (Figure 2, Table 3).

**Table 3:** Comparison of prediction performance of machine learning models.

Model	AUC (95% CI)	Sensitivity	Specificity	Accuracy	Precision	F1	Calibration Slope	Brier score
Training set								
RF	0.981 (0.968 0.994)	0.796	0.963	0.922	0.878	0.835	2.272	0.056
SVM	0.862 (0.809 0.915)	0.278	0.963	0.793	0.714	0.400	1.358	0.126
LR	0.862(0.808 0.916)	0.370	0.951	0.807	0.714	0.488	1.252	0.123
GBM	0.918(0.883 0.954)	0.611	0.920	0.843	0.717	0.660	0.719	0.103
Validation set								
RF	0.745(0.647 0.844)	0.346	0.818	0.685	0.427	0.383	0.389	0.200
SVM	0.817(0.727 0.907)	0.308	0.955	0.772	0.727	0.432	1.106	0.156
LR	0.794(0.702 0.887)	0.269	0.939	0.750	0.636	0.378	0.938	0.165
GBM	0.766(0.667 0.865)	0.385	0.788	0.674	0.417	0.400	0.719	0.174

RF, random forest; SVM, support vector machine; LR, logistic regression; GBM, gradient boosting machine.

### Evaluation of predictive performance of the optimal model

**Figure 3:**
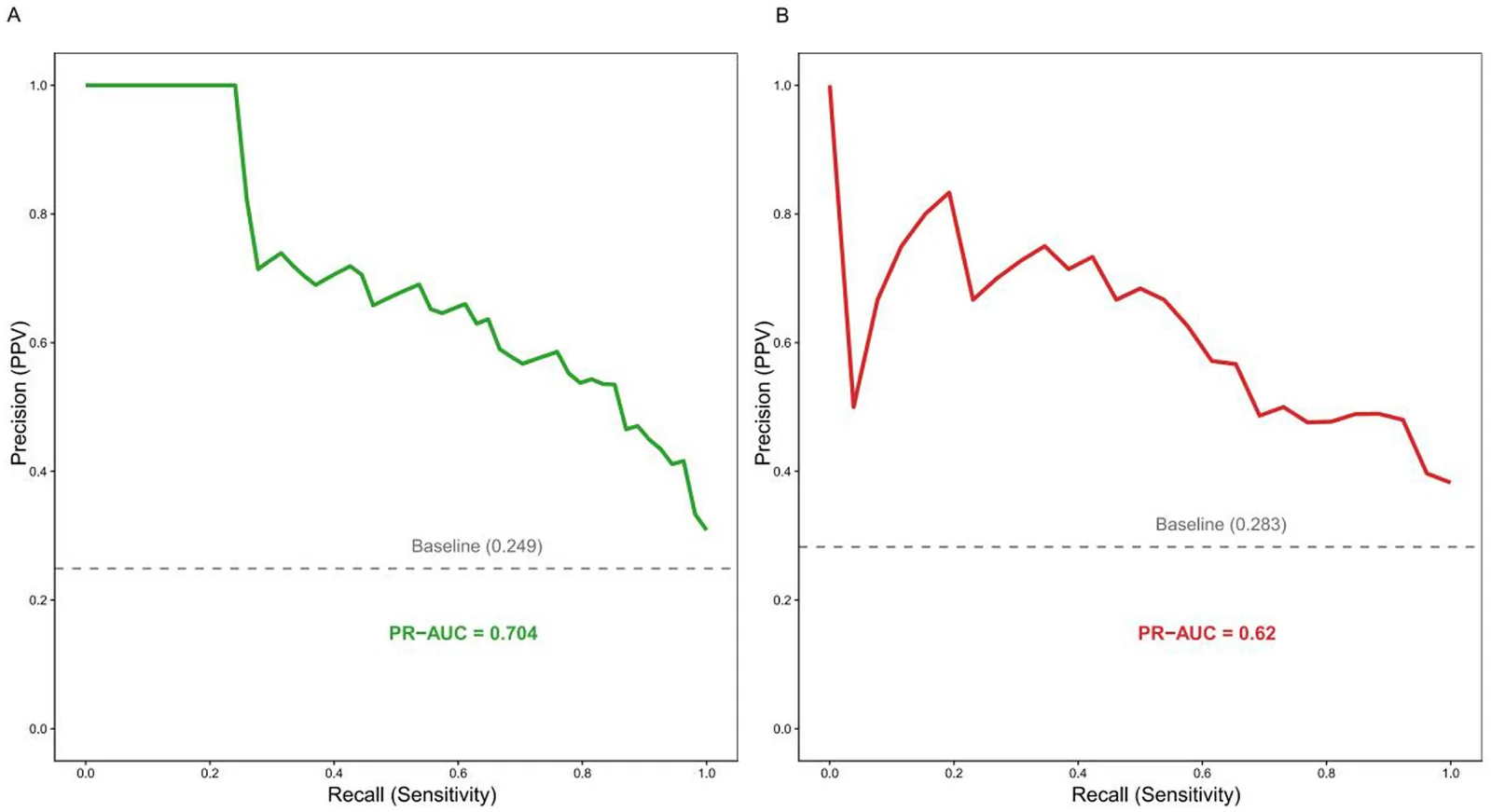
Precision-recall (PR) curve of the SVM model for predicting the transfer of patients with perioperative pneumonia exacerbation to ICU; **(A)** training set; **(B)** validation set.

The AUC of the SVM model in the training set and validation set was 0.862 (95% CI: 0.809–0.915) and 0.817 (95% CI: 0.727–0.907) respectively. An optimistic correction was conducted using the Bootstrap method (200 resampling times), and the corrected AUC was 0.816, which decreased by only about 0.17% compared to the original AUC, suggesting that the model did not have significant overfitting and had good generalization ability. Due to the imbalance of classes in the data of this study (the ICU transfer rate was 25.89%), in addition to the ROC curve, the precision-recall (PR) curve of the SVM model was drawn to better evaluate the model performance. The results showed that the PR-AUC of the SVM model in the training set and validation set were 0.704 and 0.623 respectively, both significantly higher than the baseline (0.249), indicating that the model's ability to identify ICU transfer patients was better than the random classifier (Figure 3).

**Figure 4:**
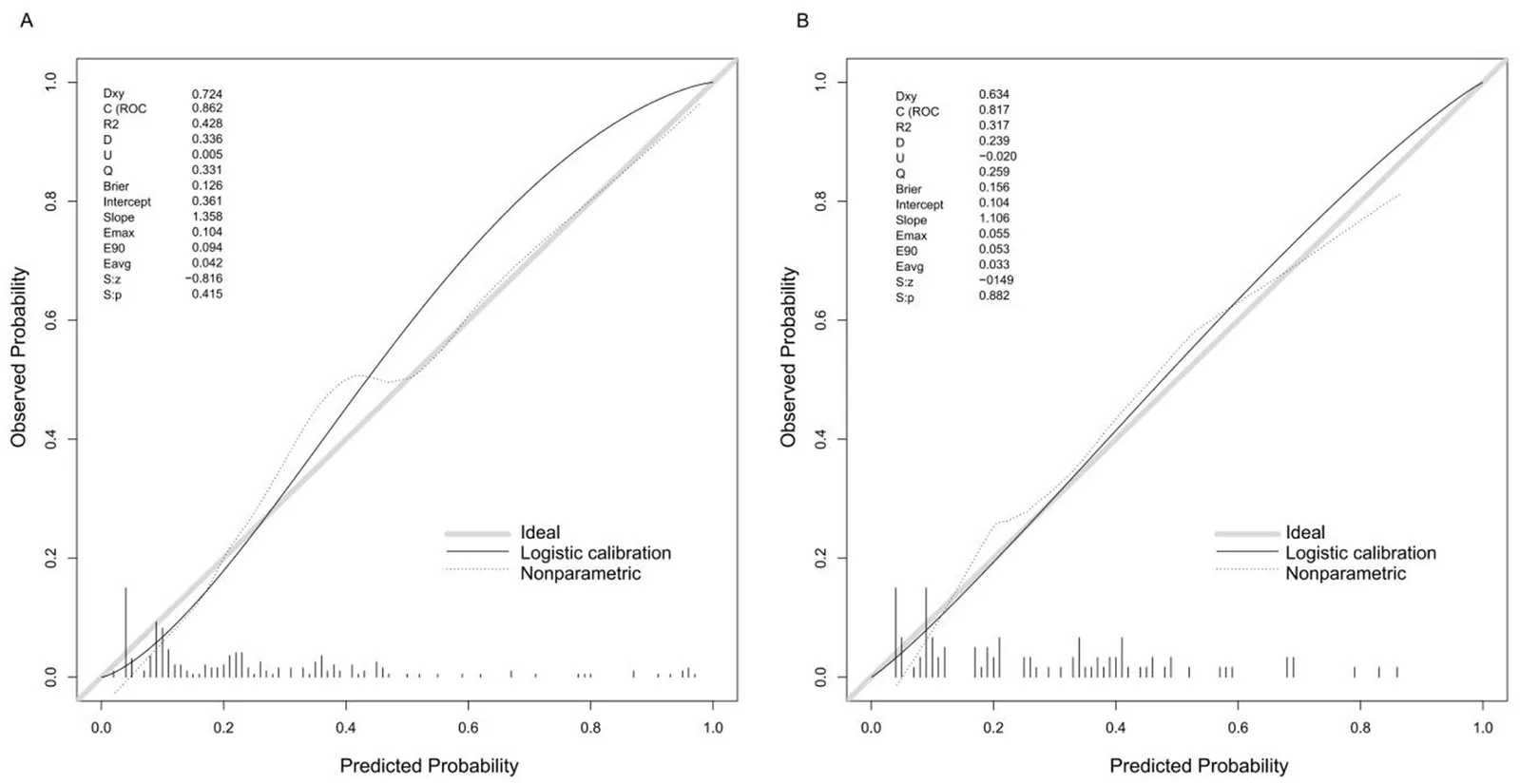
Calibration curve of the SVM model for predicting the transfer of patients with perioperative pneumonia exacerbation to ICU; **(A)** training set; **(B)** validation set.

**Figure 5:**
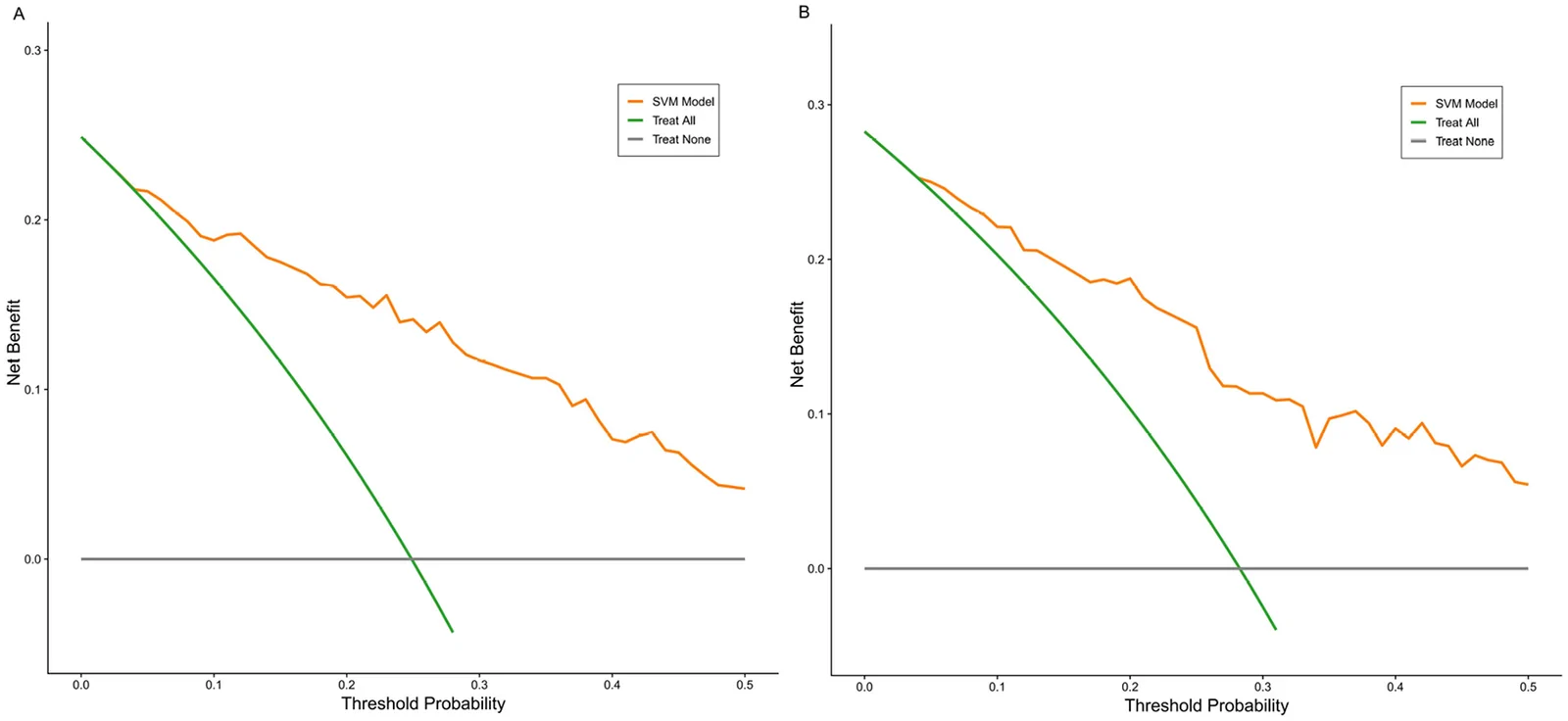
Decision curve of the SVM model for predicting the transfer of patients with perioperative pneumonia exacerbation to the ICU; **(A)** training set; **(B)** validation set.

The calibration curve showed that the predicted probabilities of the SVM model were in good agreement with the actual probabilities in both the training set and the validation set, indicating that the model had good goodness of fit (Figure 4). The DCA showed that within a risk threshold range of 0 to 0.5, intervention guided by the SVM model's predictions yielded a higher net benefit than the two extreme strategies of “intervening for all” and “intervening for none.” Among them, the net benefit of the “not intervening for any individual” strategy remains zero, while the net benefit of the “intervening for all individuals” strategy gradually decreases as the threshold increases. In contrast, the SVM model maintains a positive net benefit in a considerable portion of the threshold range. However, these results should be regarded as preliminary conclusions: the observed net benefits suggest potential clinical application value, but external validation in independent cohorts is required before recommending clinical implementation. When the threshold exceeds 0.5, the net benefit approaches zero, indicating that the value of intervention within this higher risk threshold range is limited (Figure 5).

### SHAP interpretation of the optimal model

**Figure 6:**
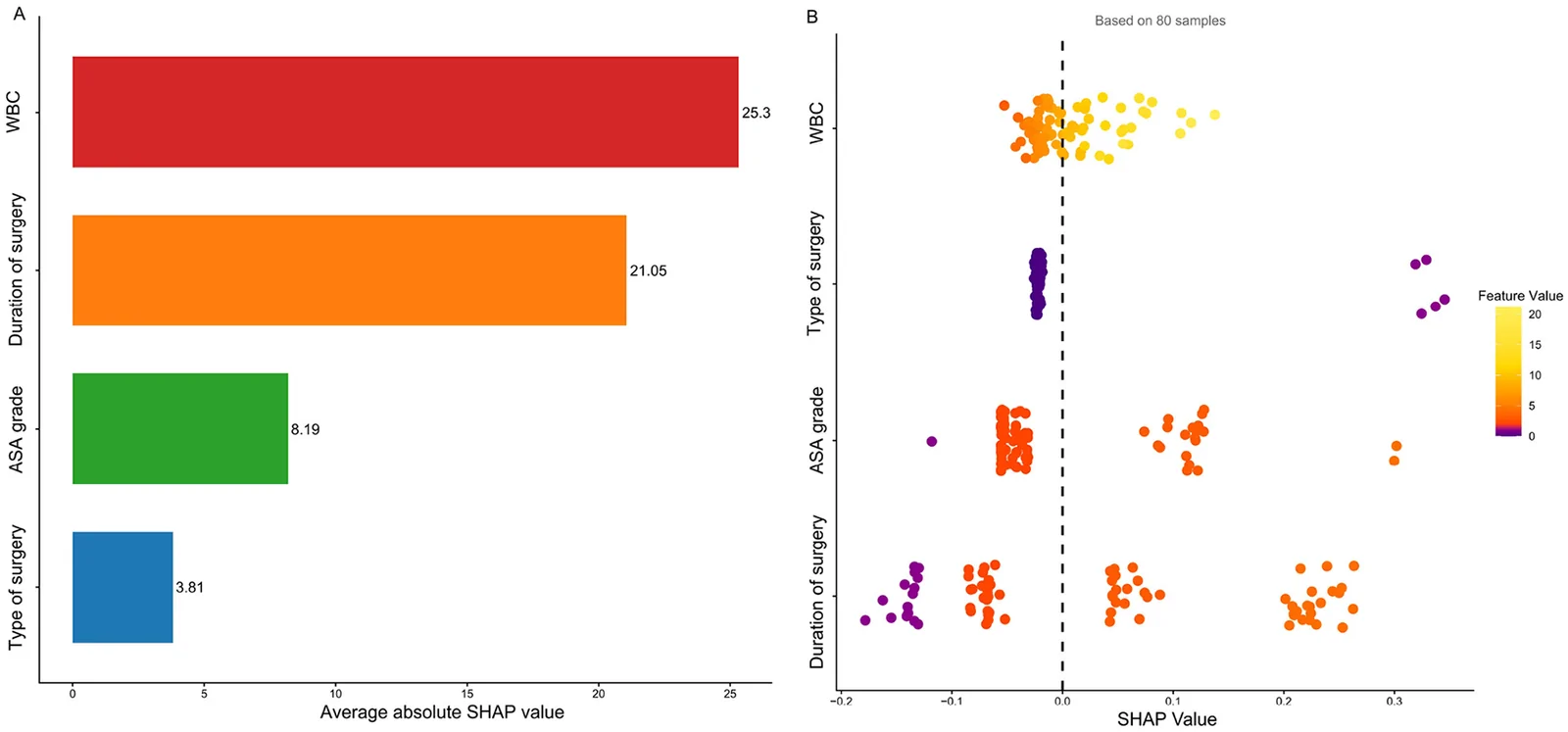
SHAP-based variable contribution analysis of the SVM model; **(A)** Mean absolute SHAP values. The Y-axis shows the variable names, and the X-axis shows the mean absolute SHAP value, representing the average contribution of each variable to the model prediction. Higher SHAP values indicate greater variable importance; **(B)** SHAP beeswarm plot. The distribution of SHAP values for each variable is shown (80 samples from the validation set). The X-axis represents the SHAP value, reflecting the direction of impact on the prediction. Color from purple to yellow corresponds to low to high feature values. Points located to the right indicate a positive contribution to the prediction of ICU transfer.

To further explain the impact of each variable on the prediction of ICU transfer in patients with worsening perioperative pneumonia, the SHAP method was used to visualize the SVM model. Figure 6A presents the mean absolute SHAP values of different variables, which can be used to measure the average contribution of each variable to the model output. The ranking from largest to smallest was as follows: WBC > duration of surgery > ASA grade > type of surgery. The SHAP summary plot (Figure 6B) showed that the prediction of ICU transfer in patients with worsening perioperative pneumonia by the SVM model was associated with a high WBC level, emergency surgery, ASA grade IV, and surgery duration >240 min.

### Individualized risk visualization based on SHAP

**Figure 7:**
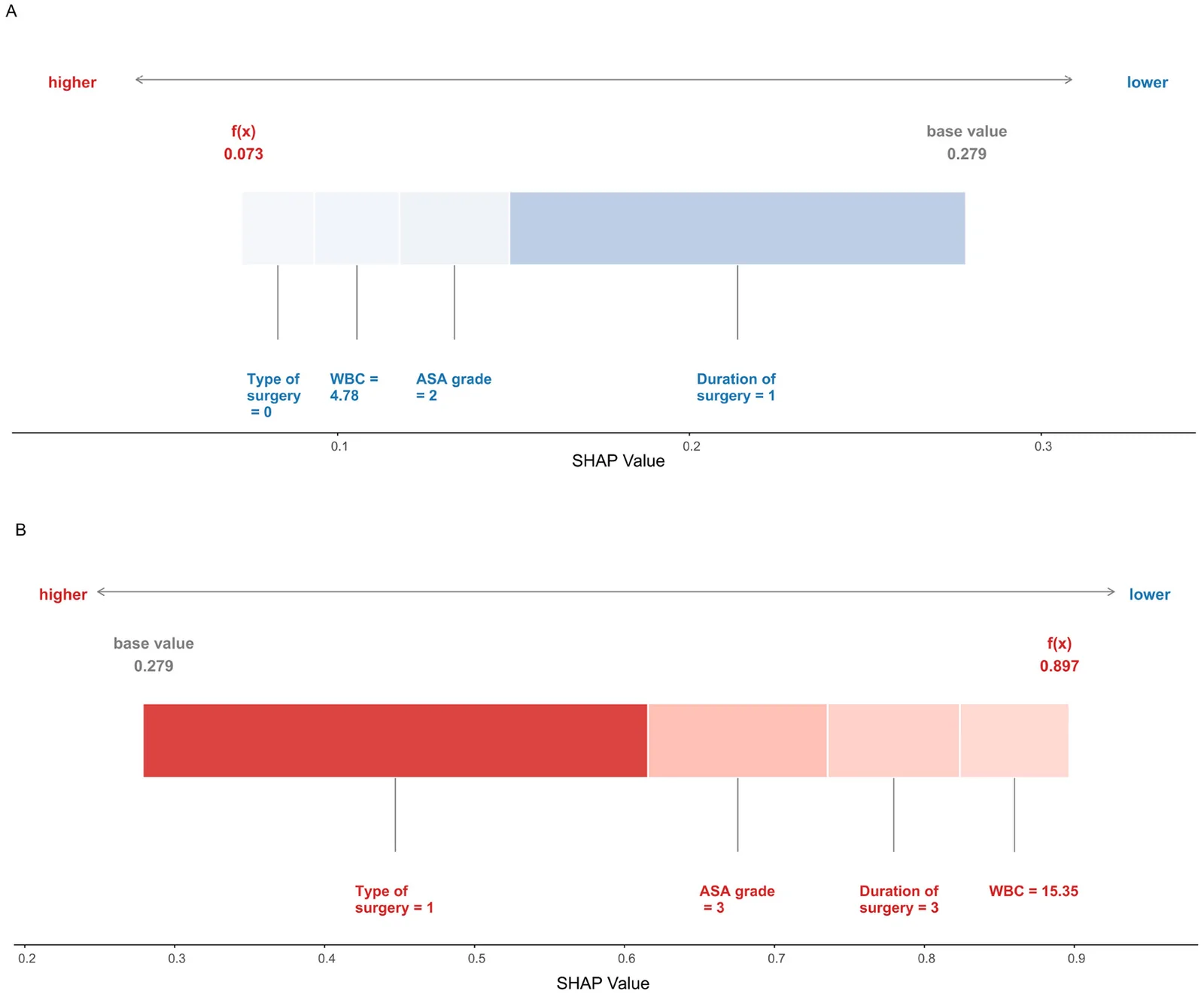
SHAP force plot for predicting the transfer of patients with perioperative pneumonia exacerbation to the ICU by the SVM model; **(A)** low-risk SHAP instance diagram based on the SVM model; **(B)** high-risk SHAP instance diagram based on the SVM model; Surgery type, Emergency = 1, Scheduled = 0; Surgery duration, 120 min=1, 120~180 min = 2, 180~240 min = 3, >240 min = 4; ASA grade, I = 1, II = 2, III = 3, IV = 4.

SHAP force plots were used to explain and visualize the risk of ICU transfer predicted by the SVM model for individual patients. One patient with worsening perioperative pneumonia who was transferred to the ICU was randomly selected. The patient had the following characteristics: Type of surgery = 1, ASA grade = 3, Duration of surgery = 3, and WBC = 15.35. The predicted risk value f(x) for ICU transfer, calculated by summing the SHAP values of the four important feature variables, was 0.897 (Figure 7B). In contrast, a randomly selected patient who was not transferred to the ICU had the following characteristics: Type of surgery = 0, WBC = 4.78, ASA grade = 2, and Duration of surgery = 1. The predicted risk value f(x) for ICU transfer was only 0.073 (Figure 7A).

## Discussion

POP is a common and serious complication of surgical procedures. Its aggravation can lead to deterioration of the patient's respiratory function, multiple organ failure, and even death (16, 17). In clinical practice, some patients with perioperative pneumonia exacerbation who progress to the condition require transfer to the ICU for further life support. This not only increases the consumption of medical resources but also significantly affects the prognosis and quality of life of patients (18). However, currently, the prediction tools for the risk of patients with perioperative pneumonia exacerbation transferring to the ICU are not yet perfect. Traditional assessment methods mostly rely on a single indicator or clinical experience, lack systematicity and quantitative standards, and make it difficult to achieve early and precise risk stratification. With the rapid development of artificial intelligence and machine learning technologies in the medical field, building risk prediction models using multi-dimensional clinical data has become an important direction in precision medicine (19, 20). Different machine learning algorithms have their own advantages in feature selection, pattern recognition, and predictive efficacy; however, for this specific group of patients with aggravated perioperative pneumonia, the algorithm with the best predictive performance and clinical applicability is currently lacking systematic comparison and verification (21, 22). Based on this, this study aimed to build a prediction model for the risk of patients with aggravated perioperative pneumonia exacerbation transferring to the ICU using multiple machine learning methods, evaluate and compare the predictive efficacy of different models, and provide an evidence-based basis for early identification of high-risk patients, optimizing perioperative management, and rational allocation of ICU resources.

This study selected four predictive variables (surgery type, surgery duration, ASA classification, and WBC) based on LASSO regression and used four machine learning algorithms (RF, SVM, LR, and GBM) to construct a prediction model for the risk of patients with perioperative pneumonia exacerbation being transferred to the ICU. The results showed that the SVM model showed the highest validation AUC in both the training and the validation sets (AUC values were 0.862 and 0.817, respectively), and the calibration curve and DCA further confirmed its good calibration and clinical net benefit. The SHAP method was used to visualize the interpretation of the model, clarifying the degree and direction of the contribution of each variable to the prediction results.

The results of this study showed that WBC count is a key factor for predicting the transfer of patients with perioperative pneumonia exacerbation to the ICU. WBC count, one of the most easily obtainable inflammatory indicators in clinical practice, can quickly reflect the intensity of the body's inflammatory response to infection. When pneumonia worsens, the body releases a large amount of inflammatory mediators, and the WBC count significantly increases, indicating an increase in the infection load and the risk of systemic inflammatory response syndrome, which in turn leads to deterioration of respiratory function and an increase in the risk of transfer to ICU (23). Xu et al. (24) in a systematic review on machine learning in the prediction of post-acute infection found that inflammatory-related indicators were one of the core factors for individual-level risk prediction and were closely related to the severity of infection and adverse prognosis. Xiang et al. (2) also confirmed a postoperative pneumonia prediction model based on nine machine learning algorithms, which also demonstrated that inflammatory-related indicators were important predictive variables of the model. These evidence indicate that WBC count, as a direct manifestation of the inflammatory response, has significant value in assessing the severity of perioperative pneumonia and predicting the risk of transfer to the ICU.

This study found that the duration of surgery was an independent predictor of patient transfer to the ICU, ranking second in the SHAP feature importance ranking. Extended surgical duration implies an increase in anesthesia exposure time, aggravated intraoperative stress response, and increased difficulty in respiratory management. These factors can lead to cumulative lung function impairment, increasing the risk of perioperative pneumonia exacerbation and deterioration of the condition (25, 26). The development and validation results of the international multicenter GSU-Pulmonary Score study showed that the duration of surgery is an important predictor of postoperative pulmonary complications. The predictive model constructed based on 86,231 patient data in this study covered key variables, such as the duration of surgery (1). Additionally, the nomogram prediction study on pulmonary infection after radical gastrectomy by Xiao et al. (27) demonstrated that a surgery duration of ≥ 4 h was a significant predictor of postoperative pulmonary infection, and the proportion of infected patients requiring ICU treatment was significantly higher than that of non-infected patients (23.08% vs. 1.83%, *P* < 0.001), which was highly consistent with the outcome indicators of this study, further confirming the significant impact of the duration of surgery on the aggravation of perioperative pneumonia and the risk of ICU transfer. For high-risk patients with a long surgery duration, respiratory management should be strengthened after surgery, and infection indicators should be closely monitored. Early intervention should be conducted when necessary to prevent disease progression.

The ASA classification, as a classic indicator for evaluating the preoperative overall health status of patients, has been confirmed by multiple studies to be closely related to postoperative adverse outcomes. This study found that an elevated ASA classification significantly increased the risk of patients with perioperative pneumonia exacerbation being transferred to ICU, ranking third in the SHAP feature importance. Hackett et al. (28) based on the NSQIP database and including 2,297,629 patients in a large sample study showed that ASA classification was an independent predictor of postoperative medical complications and death. When the ASA classification increased from level 2 to level 5, the OR for complications and mortality increased from 2.05 to 63.25 and 5.77 to 2011.92 (both *P* < 0.001). After standardization by surgical type (Z-score), the complication risk still increased by 42% for every 1 standard deviation increase in ASA classification. This is consistent with the high ASA classification, which has more comorbidities, decreased physiological reserve, and reduced stress compensation ability. Laporta et al. (29) conducted a study on non-cardiothoracic surgery patients and confirmed that ASA classification has a significant dose-response relationship with postoperative pneumonia risk. The OR values of pneumonia risk for ASA III and > III patients were 4.17 (95% CI: 1.74–10.01) and 24.03 (95% CI: 6.54–88.32), respectively. This may be because that patients with high ASA classification have more comorbidities, decreased physiological reserve, and reduced stress compensation ability, making them more prone to infection and organ failure. Therefore, for patients with a high ASA classification, adequate respiratory function optimization should be performed before surgery, precise operations during surgery to reduce trauma, and enhanced postoperative monitoring to identify early deterioration of the condition.

Surgery type (emergency vs. elective) ranked highly in the feature importance ranking, suggesting that surgical timing significantly impacts the prognosis of patients with perioperative pneumonia exacerbation. Wu et al. (30) demonstrated in a large-scale retrospective study of 399,347 surgical patients that the incidence of postoperative pneumonia (POP) in emergency surgery (2.28%) was significantly higher than that in elective surgery (0.53%, P < 0.001), with emergency surgery carrying more than a 4-fold increased risk of POP. Multivariate analysis further identified male sex, age ≥60 years, admission to ICU, ASA grade ≥3, general anesthesia, surgical duration ≥3 h, and intraoperative blood loss >1,000 mL as independent risk factors for POP in emergency surgery. Ferrando-Ortolá et al. (31), in the prospective multicenter international PEAL study of 507 patients who underwent emergency abdominal surgery, reported a postoperative pulmonary complication (PPC) rate of 22.5%, with severe PPCs accounting for 7.5%, further confirming the elevated risk in this population. These findings suggest that clinicians should establish more proactive perioperative management protocols in patients undergoing emergency surgery and strengthen postoperative respiratory monitoring to enable the early detection of deterioration.

This study compared the predictive efficacies of four machine learning algorithms. In the validation set, the SVM model achieved the highest AUC (0.817), followed by LR (0.794), GBM (0.766), and RF (0.745). However, DeLong test showed that the AUC difference between SVM and LR was not statistically significant (*P* > 0.05), indicating that the apparent advantage of SVM over LR in this dataset was modest and may be influenced by random variation. SVM effectively maps low-dimensional features to high-dimensional spaces through the kernel function and can handle small sample sizes and non-linear problems, which may explain its relatively stable performance in this study (309 cases). A large-scale cohort study involving 111,888 surgical patients showed that when using machine learning models, such as gradient boosting trees (GBT), SVM, and RF, to predict postoperative pneumonia and other complications, the SVM model also demonstrated good predictive efficacy, with a pneumonia prediction AUC of 0.905 (32). Another study on the prediction of postoperative pneumonia after liver transplantation compared six machine learning algorithms. The XGBoost model performed the best (AUC = 0.734), and the SVM also had a good predictive ability (33). The LR model performed slightly worse than SVM in this study, possibly due to its assumption of linear relationships among features. RF and GBM, as ensemble learning models, may have overfitting risks in small sample situations, which can be confirmed by the greater decrease in AUC from the validation set to the training set compared to SVM. Notably, the validation-set sensitivity of the SVM model was only 0.308 at the default threshold (0.5). This means that the model could identify only approximately one-third of patients who would truly require ICU admission, while missing approximately two-thirds of actual ICU-transfer cases. This result is closely related to our threshold selection strategy, which prioritized specificity (0.955) to avoid unnecessary ICU admissions. However, this low sensitivity substantially limits the model's value as a stand-alone screening tool. In its current threshold configuration, the model is more suitable as a supportive reference for clinical decision-making than as an independent early-warning system. Future work should consider optimizing the risk threshold, introducing cost-sensitive learning, or incorporating dynamic monitoring data to achieve a better balance between sensitivity and specificity and may improve detection in future research.

This study has the following limitations: (1) It was a retrospective single-center study, which may have selection and information biases. Some perioperative-related indicators (such as intraoperative fluid management, use of vasoactive drugs, mechanical ventilation parameters, etc.) were not included in the analysis, which may affect the comprehensiveness of the model; Furthermore, the model has a relatively low sensitivity under the default threshold, which is not only related to the threshold setting strategy (prioritizing specificity), but also reflects the limitations of the model in identifying high-risk patients. Its predictive performance may be further improved through threshold optimization in future studies. (2) The sample size is relatively limited (309 cases). Although the training and validation sets were divided in a 7:3 ratio, no external independent dataset was introduced for validation, and the extrapolation and generalization ability of the model remains to be established in external cohorts; (3) The definition and diagnostic criteria for perioperative pneumonia exacerbation may vary in different research centers. This study was based on a single-center clinical practice, and the conclusions should be interpreted with caution when applying to other institutions; (4) This study employed the SMOTE algorithm to address class imbalance. It was applied only within the cross-validation folds to avoid data leakage. However, synthetic data may introduce certain biases. The stability of the model should be further verified using real external data; and (5) This study did not conduct time validation; thus, it could not assess the stability of the model over time. Future multicenter prospective studies with larger sample sizes are needed to validate these findings. Additionally, incorporating more potential predictive variables (such as inflammatory profiles, radiomics features, or genomic markers) and exploring deep learning approaches with time-series data (e.g., vital sign trends, dynamic laboratory changes) may further improve prediction accuracy.

In summary, this single-center study developed and internally validated prediction models for ICU transfer in patients with perioperative pneumonia exacerbation using four machine learning algorithms. Among them, the SVM model showed the highest validation AUC, but its low sensitivity at the default threshold limits its potential as a screening tool in this dataset. WBC count, duration of surgery, ASA grade, and type of surgery were identified as key risk factors. External validation in multicenter cohorts is needed before any clinical application can be considered.

## Data Availability

The original contributions presented in the study are included in the article/supplementary material, further inquiries can be directed to the corresponding authors.
